# Planning a Clinical Practice Guideline Revision for a Core Set of Outcome Measures in Adults Undergoing Neurologic Physical Therapy, Part 1: Assessing Dissemination and Scholarly Uptake

**DOI:** 10.1002/gin2.70086

**Published:** 2026-09-06

**Authors:** Jennifer L. Moore, Jane E. Sullivan, Marghuretta Bland, Wendy Romney, Elizabeth Anderl, Zeynep Gulen Erkoc, Kirsten Potter

**Affiliations:** ^1^ Institute of Knowledge Translation, and Regional Rehabilitation Knowledge Center Sunnaas Rehabilitation Hospital Oslo Norway; ^2^ Department of Physical Therapy and Human Movement Sciences Feinberg School of Medicine, Northwestern University Evanston Illinois USA; ^3^ Washington University School of Medicine Program in Physical Therapy St. Louis Missouri USA; ^4^ Department of Physical Therapy and Human Movement Science Sacred Heart University Fairfield Connecticut USA; ^5^ TIRR Memorial Hermann Houston Texas USA; ^6^ Groton School Groton Massachusetts USA; ^7^ School of Medicine, Department of Rehabilitation Sciences, Doctor of Physical Therapy Tufts University Seattle Washington USA

## Abstract

**Introduction:**

Clinical Practice Guidelines (CPGs) facilitate evidence‐based decision‐making and standardising care, yet their dissemination and scholarly uptake vary widely. The Core Set of Outcome Measures for Adults With Neurologic Conditions Undergoing Rehabilitation CPG (Core Set) has been broadly referenced within neurologic physical therapy. As the next revision approaches, understanding its scholarly influence, citation patterns, and published feedback is essential. This study evaluated the dissemination and use of the Core Set across the peer‐reviewed literature and identified areas for refinement.

**Methods:**

A medical librarian conducted comprehensive searches of Web of Science, CINAHL, and Scopus to identify peer‐reviewed English‐language articles citing the Core Set between July 1, 2018, and July 31, 2024. Reviewer pairs screened articles and extracted publication characteristics, citation context using an adapted Rabin et al. and Field et al. framework, and narrative feedback. Quantitative data were summarised descriptively. Constructive and positive feedback were thematically coded. Altmetric Attention Score, PlumX captures, and Mendeley readership data characterised broader dissemination and engagement.

**Results:**

Of 388 records, 176 articles met inclusion criteria. Most articles cited the Core Set once (*n* = 106), most frequently in the introduction (*n* = 70) or methods (*n* = 65). Thirty‐two percent (*n* = 57) reported that the Core Set informed study design, guided outcome measure selection, or was implemented in practice. Studies represented 30 countries, with the United States contributing the largest proportion (*n* = 104). Constructive feedback (14 articles) highlighted needs related to standardisation procedures, diagnosis‐specific applicability, construct coverage, and feasibility. Positive feedback (9 articles) emphasised methodological clarity, usability, and clinical value. Dissemination indicators demonstrated broad engagement, including an Altmetric Attention Score of 60, 261 scholarly citations, 643 PlumX captures, and 604 Mendeley readers.

**Discussion:**

Findings support maintaining the Core Set's methodological rigour while providing some suggestions for improvement.

## Summary

- This study assessed scholarly dissemination, citation patterns, citation context, and published feedback related to the 2018 CPG to inform the upcoming CPG revision.
- Of 388 records, 176 articles met inclusion criteria. In 57 articles, the 2018 CPG informed or was integrated into the study, or its recommended outcome measures were implemented in practice. Studies were conducted in 30 countries, as well as one international multi‐site study spanning 18 countries.
- Constructive feedback addressed standardisation procedures, diagnosis‐specific limitations, construct‐related concerns, and feasibility and use of the recommendations. Positive feedback emphasised the value of the measures and recommendations provided in the 2018 CPG for research and practice.
- Dissemination indicators demonstrated broad engagement, including an Altmetric Attention Score of 60, 261 scholarly citations, 643 PlumX captures, and 604 Mendeley readers. These metrics do not indicate whether the document was read, considered useful, adopted, or implemented in practice.

## 1 Introduction

Clinical Practice Guidelines (CPGs) are systematically developed statements intended to assist practitioners and patients in making informed decisions about appropriate healthcare, based on comprehensive reviews of evidence and evaluations of the relative benefits and risks of available care options [1, 2]. These documents are critical to support the implementation of evidence‐based practices and standardisation of healthcare [2]. They offer practitioners standardised approaches to patient care derived from the latest research findings and expert consensus. They also guide clinical decision‐making, examination and treatment strategies, and healthcare policies across various medical specialties [3]. CPGs also have a significant role in informing research in several ways by identifying research gaps, setting research agendas, and validating or challenging existing research [3].

The Core Set of Outcome Measures for Adults Undergoing Neurologic Physical Therapy CPG used a rigorous process of evidence synthesis and expert consensus to recommend a group of specific measures each assessing specific constructs of clinical practice [4]. Although related to core outcome set initiatives, this group of specific measures was developed as a clinical practice guideline rather than as a formal core outcome set developed using methodology from the Core Outcome Measures in Effectiveness Trials initiative [5]. In this paper, we use the capitalised “Core Set” to refer to the CPG and its recommended set of outcome measures. By identifying a specific group of outcome measures tailored to the unique needs of individuals with neurological conditions, the Core Set aimed to standardise the use of outcome measures to assess patient change over time, facilitate comparison of patient outcomes, promote the use of evidence‐based practice, and enhance quality of care and documentation. The Core Set included detailed recommendations for measure selection, administration, and interpretation, as well as guidance for clinical implementation. As with all CPGs, regular updates are essential to ensure that recommendations reflect the most current evidence [6].

While there are no universally accepted best practices for evaluating the impact of a CPG, evaluation generally focuses on its dissemination and implementation in practice [7]. Dissemination has been defined as the targeted distribution of information and intervention materials to a specific public health or clinical practice audience [8], whereas clinical impact reflects how consistently and appropriately it is applied in practice [7, 9]. Dissemination can be evaluated through citation analysis, which reflects the extent to which a CPG informs subsequent research, as well as through awareness and reach indicators such as clinician surveys, distribution records, and website access statistics [7, 10]. Altmetric indicators, including the Attention Score, mentions across news, social media, and policy documents, provide insight into the visibility of a CPG across digital platforms [11]. PlumX Metrics and Mendeley Readership Counts reflect the frequency that users save or bookmark a CPG for future reference [11, 12]. Usage metrics, such as full‐text views, PDF downloads, and webpage analytics, provide further insight into the frequency and breadth with which a CPG is accessed or saved. However, these metrics do not indicate whether the document was read, considered useful, adopted, or implemented in practice [7, 10].

In contrast, clinical impact reflects whether a CPG influences care delivery or patient outcomes. Approaches for evaluating clinical impact include assessing the degree of adherence to CPG recommendations in routine care, impact on patient outcomes and health systems, and variables such as perspectives and attitudes related to the CPG [13]. Studies evaluating clinical impact vary widely in methodological rigour, and many rely on surrogate outcomes or incomplete measures of adherence, making it difficult to determine the true effect of CPG use on patient health [14]. Together, these complementary approaches offer a comprehensive view of CPG impact. Dissemination metrics, including citation and usage data, capture the reach of a guideline, whereas clinical implementation studies reveal its effect on care delivery and patient outcomes.

The purpose of this study was to assess scholarly dissemination, citation patterns, citation context, and published feedback related to the Core Set to inform the upcoming CPG revision. Specifically, we aimed to (1) examine citation patterns over time, (2) analyze how the Core Set was referenced in subsequent research, (3) synthesise narrative feedback to inform potential areas for clarification, expansion, or refinement in the revision, and (4) evaluate article‐level metrics as supplementary indicators of scholarly visibility and reach.

## 2 Methods

This study was conducted as a bibliometric citation analysis with descriptive content analysis of citation context and narrative feedback. A medical librarian conducted structured cited‐reference searches in Web of Science, CINAHL, and Scopus to identify peer‐reviewed publications citing the Core Set between July 1, 2018, and July 31, 2024. These databases were selected because they provide cited‐reference indexing and complementary coverage of rehabilitation and health sciences literature. Web of Science was selected because of its long‐standing cited‐reference indexing and broad cross‐disciplinary coverage. Scopus was included to expand citation tracking across a large multidisciplinary database, and CINAHL was included to improve capture of rehabilitation, nursing, and allied health journals relevant to physical therapy practice. Because this study focused on peer‐reviewed scholarly dissemination and citation context, grey literature sources were not searched. No additional backward or forward snowballing outside these databases was performed. The search cutoff date of July 31, 2024, reflected the timeframe during which the Core Set Revision Guideline Group was actively conducting preparatory work for the upcoming CPG revision and aligned with the timeline required to synthesise findings and inform revision planning decisions. Search results from each database were exported, combined, and deduplicated using EndNote and manual review before screening. Articles were included if they (1) cited the Core Set, (2) were peer‐reviewed, and (3) were published in English. No additional filters or limits were used. The reviewers consisted of the six members of the Core Set Revision Guideline Group (JLM, KP, JES, WR, MDB, EA). To promote consistency across reviewers, all reviewers initially screened, reviewed, and extracted data from 10 articles. Conflicts identified during this process were discussed and resolved by the full group. These discussions were also used to refine and clarify our screening and data extraction procedures.

The titles, abstracts, and full texts were screened by pairs of reviewers to confirm eligibility. Study selection is summarised in a PRISMA‐style flow diagram (Figure 1). Full‐text review and data extraction were conducted by the reviewer pairs. The extracted variables included publication details (title, journal, date, country), corresponding author location, study design, citation context, citation frequency, article section of citation(s), diagnosis, setting, and disciplines involved (Table S1). The citation context was categorised into predefined types (e.g., referenced, directed, integrated, implemented) using a framework adapted from Rabin et al. (2022) and Field et al. (2014) [15, 16]. Definitions and examples of the context are in Table 1. When articles included narrative commentary on the Core Set, quotations were extracted and coded as either constructive or affirming feedback.

**Figure 1:**
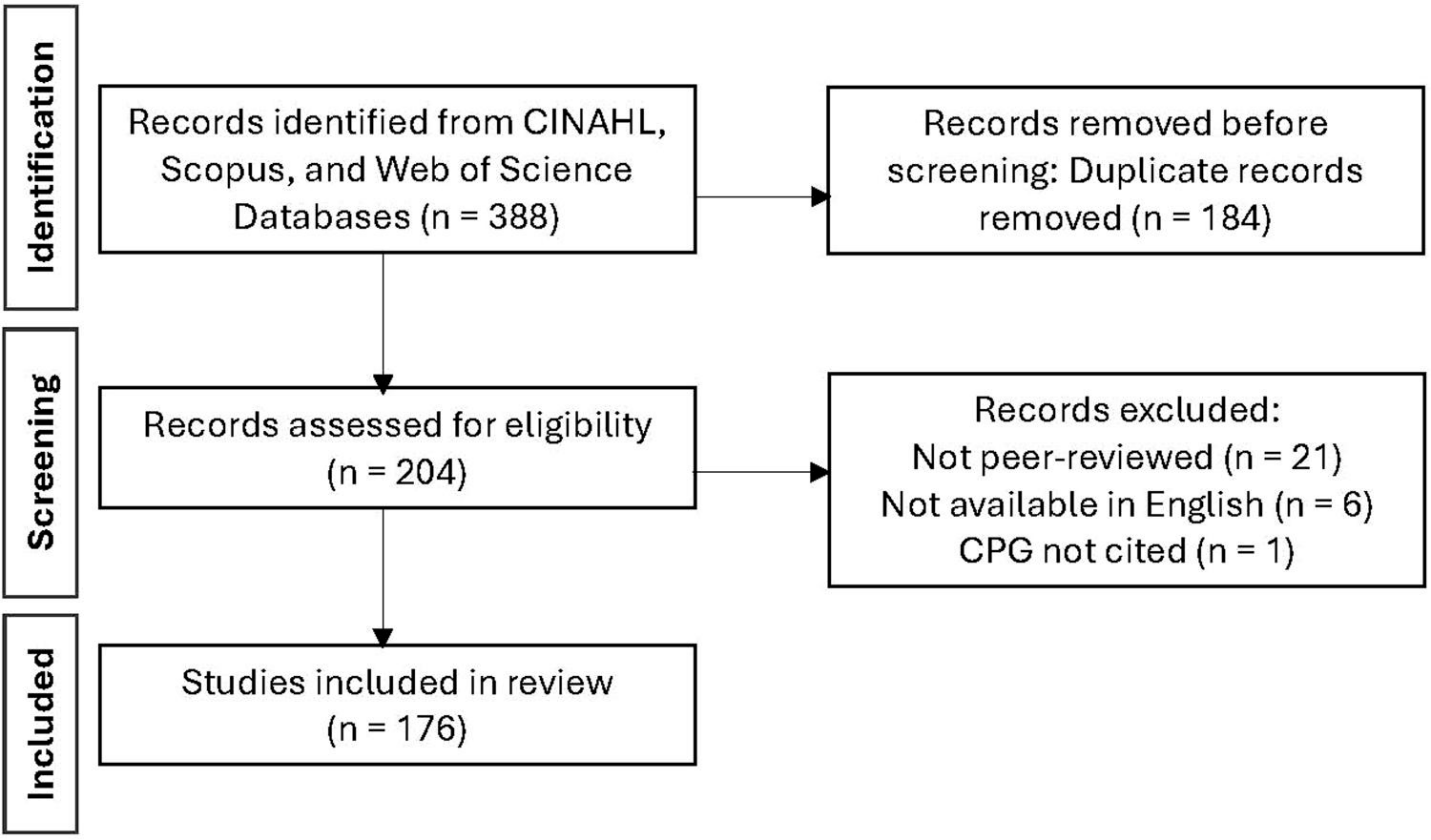
Flow diagram for included studies.

**Table 1:** Core set citation context within each article and the number of times that citation context was used.

Type of use	Definition	Examples or Descriptions	Citation Count *n* = 186	Percent (citations/186)
Framing Field/Area	The Core Set was used to understand the current state of the field and guide direction for future research and practice.	“Recently, the ABC, 5TSTS, and 10MWT have been identified as core measures to use with patients with neurologic conditions across practice settings who have the capacity and ability to improve balance confidence, transfers, and gait [17].”	61	33%
Review	The Core Set was used in a review of research as one of the articles reviewed.	For example, see Dos Santos et al., 2023 [18].	2	1%
Referenced	The Core Set was cited to establish a broader context for the field with no further explanation of direct relevance to the CPG.	“Outcome measurements are an important component of neurological rehabilitation programmes [19].”	62	33%
Informed	The Core Set was cited and named often in the introduction or discussion as it related to the study or future studies but no explanation of direct use.	“Balance and gait evaluations are considered as key features in this assessment [20].”	1	1%
Directed	The Core Set was cited in the methods and influenced the project planning or design	“Demographics and baseline measures of motor and cognitive function were collected to characterise the sample, including Montreal Cognitive Assessment, self‐selected and fast gait speeds via the 10 Metre Walk Test [21]…”	55	30%
Integrated	The Core Set was integral to the planning, design, delivery, evaluation, interpretation, and/or dissemination of the project/study.	This is an appropriate category for studies that aimed to address gaps in the Core Set. For example, a primary aim is related to testing protocols, psychometrics, etc., with the rationale being related to information in the Core Set or associated task force documents. For example, see discussion in Kahn et al, 2020 [22] or Bland et al, 2024 [23]	4	2%
Implemented in practice	At least one measure from the Core Set was implemented in clinical practice as a component of a knowledge translation project. The Core Set was cited as the rationale for implementing the measure.	For example, see Yorke et al., 2021 [17].	1	1%

*Note:* The core set was cited more than once per article in some articles.

Data were extracted by two reviewers and separately logged in a form in Airtable (Airtable, San Francisco, CA, USA, https://www.airtable.com/), a cloud‐based database platform that allows for real‐time collaboration, customisable fields, and data organisation across multiple reviewers. A research assistant (ZGE) identified conflicts between the two reviewers and missing data. When conflicts were identified, the reviewer pair evaluated and modified their responses if needed. Any remaining conflicts were resolved through discussion and consensus.

### 2.1 Analysis

Descriptive statistics were computed using Microsoft Excel. Pivot tables were used to summarise quantitative variables such as citation frequency, study design, practice setting, diagnoses, disciplines, and geographic location. An inductive coding approach was used to identify emergent themes from the feedback data. Citation contexts and narrative feedback were independently reviewed by two authors (J.L.M. and W.R.), who then collaboratively agreed on themes and coded the data. Constructive feedback themes include standardisation procedures, diagnosis, measure addition or substitution, construct, setting, feasibility, and use of the Core Set. Altmetric Attention Scores and Mendeley reader counts associated with the Core Set were obtained using Altmetric, PlumX, and Mendeley records linked to the article as of October 27, 2025 to evaluate broader dissemination [11, 12, 24].

## 3 Results

A total of 388 articles were identified through database searches. After removing 184 duplicates and excluding 28 articles that did not meet eligibility criteria, 176 peer‐reviewed articles published between July 1, 2018, and July 31, 2024, were included in the final citation analysis. Excluded records consisted of non‐peer‐reviewed publications (*n* = 21), articles not available in English (*n* = 6), and one article that did not cite the Core Set in the full text. Figure 1 describes the search results.

Citations of the Core Set started with one in 2018 and peaked in 2022 with 46 citing articles. The median number of citations per year was 25 (IQR, 18.5–33.5). As shown in Figure 2 citations almost doubled from 2021 to 2022 and then less rapidly declined. Most articles cited the Core Set once (*n* = 106), with fewer citing it twice (*n* = 38) or three or more times (*n* = 32). Citations appeared most frequently in the introduction (*n* = 70), followed by the methods section (*n* = 65) then discussion (*n* = 48). As shown in Table 1, the context and purpose of Core Set citations varied across articles. The guideline was often cited to provide background context for the field (*n* = 62), to frame the research area and inform future directions (*n* = 61), or to guide study design and methodology (*n* = 55). The research designs of citing articles are provided in Table S2 and include psychometric studies (*n* = 49, 28%), cohort studies (*n* = 31, 18%), case studies (*n* = 19, 11%), and systematic reviews, meta‐analyses or CPGs (*n* = 16, 9%). Of the articles citing the Core Set, neurologic populations were the primary focus in 140 articles, with stroke (*n* = 57), multiple sclerosis (*n* = 21), and Parkinson's disease (*n* = 14) emerging as the most frequently studied conditions. Additional clinical populations included geriatrics (*n* = 14), cardiopulmonary (*n* = 5), musculoskeletal (*n* = 4), paediatrics (*n* = 3) and oncology (*n* = 1). Articles described research conducted in outpatient setting, including clinical and research environments, most frequently (*n* = 74, 54%), followed by inpatient rehabilitation, subacute rehab, and skilled nursing facilities (*n* = 37, 27%). Other studies were conducted in community settings where individuals were not actively receiving rehabilitation (*n* = 10, 7%), acute or intensive care units (*n* = 9, 7%), and home health (*n* = 7, 5%). Table 2 provides an overview of areas of practice, settings, and medical diagnoses of articles referencing the Core Set.

**Figure 2:**
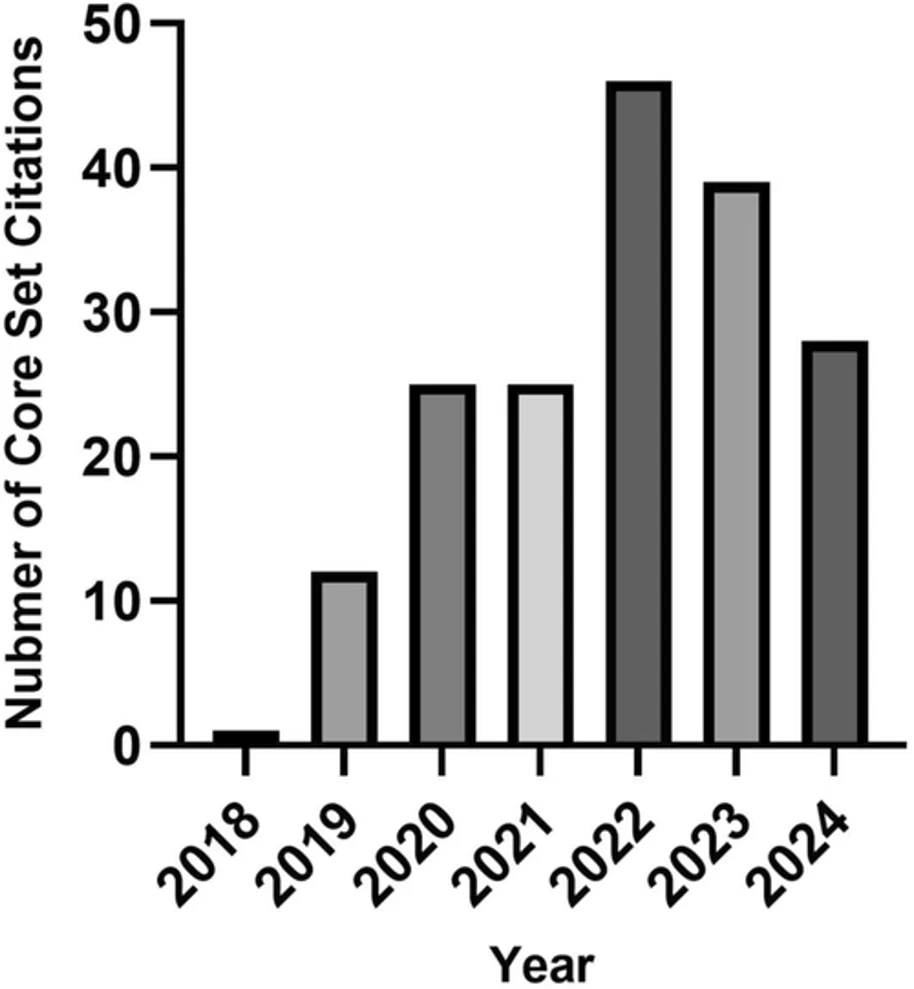
Citation patterns over time.

**Table 2:** American Physical Therapy Association (APTA) clinical specialty areas of practice and medical diagnosis of articles referencing the Core Set. Some manuscripts had multiple diagnoses or areas of practice.

APTA clinical specialty areas of practice	Citation count 191	Percent
Neurology	140	73%
Geriatrics	14	7%
Cardiovascular and Pulmonary	5	3%
Orthopaedics	4	2%
Paediatrics	3	2%
Oncology	1	1%
Other Area of Practice^a^	24	13%
**Setting**	**137**	
Outpatient (includes clinical, research studies)	74	54%
Inpatient (Inpatient rehabilitation, sub‐acute rehabilitation, skilled nursing facility, etc.)	37	27%
Community (not receiving services)	10	7%
Acute or Intensive Care Unit	9	7%
Home Health	7	5%
**Medical Diagnosis**	**154**	
Stroke	57	37%
Multiple Sclerosis	21	14%
Parkinson's disease	14	9%
Non‐specific	10	6%
Non‐specific neurologic	9	6%
Acquired brain injury	8	6%
Older adults	4	3%
Cerebellar dysfunction	3	2%
Cognitive impairment	3	2%
COVID‐19	3	2%
Guillain‐Barré Syndrome	3	2%
Neuropathy	2	1%
Post‐Intensive Care Syndrome	2	1%
Other^b^	15	10%

a Other Area of Practice (Count, Percent): Healthy Adults (7, 4%), Acute Care (5, 3%), General Rehabilitation (2, 1%), Chronic Pain (1, 1%), COVID (1, 1%), Dietetics (1, 1%), General (1, 1%), Knowledge Translation (1, 1%), Multiple diagnoses (1, 1%), Non‐Specific (1, 1%), Nutrition (1, 1%), Obesity (1, 1%), Prosthetics (1, 1%), Public Health (1, 1%), University Student (1, 1%).

^b^ Other Medical Diagnoses each with a count of 1, 1%: Amputee, Amyotrophic Lateral Sclerosis, Arthrogryposis Multiplex Congenita, Charcot‐Marie Tooth Disease, Chronic Obstructive Pulmonary Disease, Functional Neurologic Disorder, Heart Failure, Hip Fracture, Huntington's Disease, Lymphoma, Normal Pressure Hydrocephalus, Obesity, Patients receiving dietetic services, Susac Syndrome, Vestibular Disorders.

Studies citing the Core Set were conducted in 30 countries, as well as one international multi‐site study spanning 18 countries. Most publications originated in the United States (*n* = 104), followed by Brazil (*n* = 7), and Japan (*n* = 6). Among these articles, 57 indicated that the Core Set informed or was integrated into the study, or that its recommended outcome measures were implemented in practice. See Table S3 for more details.

Constructive feedback on the Core Set was provided in 14 of 176 articles, (Table 3). The most common feedback theme was related to standardisation procedures (4 articles; 5 feedback instances), with concerns raised about the reliability and comparability of the 6‐min Walk Test [27, 44, 45]. Feedback also addressed the standardisation of the Five Times Sit‐to‐Stand test, specifically the number of recommended trials [28]. Other feedback focused on diagnosis‐specific limitations (*n* = 4), such as the applicability of the measures in patients with higher levels of function and those who have multiple sclerosis, Guillain‐Barré Syndrome, and concussion [29, 30, 31, 32]. Other suggestions included adding [31] or substituting measures [31, 33]. Construct‐related concerns indicated that the measures recommended in the Core Set may assess capacity rather than actual performance [34] and may not represent community independence [35]. Some authors questioned the Core Set's utility in home health [36] or the community [35]. Lastly, feedback was provided about the feasibility and use of the recommendations provided in the Core Set. Some authors indicated there were too many measures, in addition to already required measures [37] and the 6‐min Walk Test is too long [30]. Additionally, authors noted a need for more guidance on how to administer measures that assess overlapping constructs [38].

**Table 3:** Summarised constructive and positive feedback provided about the Core Set.

Constructive feedback theme	Citation Count 14	Constructive feedback
Standardisation Procedure	5	Reliability of 10MWT is insufficient for all individuals with stroke [25]. Standardisation protocol of 6MWT is shorter, results in more turns, less distance than other protocols [26, 27]. Recommended mathematical calculation of distance for 6MWT instead of measuring wheel [26]. Standardisation protocol of 6MWT is shorter than American Thoracic Society guidelines, which limits ability to compare results [26]. Standardisation procedure for 5xSTS questioning the recommended number of sit‐to‐stand trials [28].
Applicability in specific diagnoses	4	Guillain‐Barré Syndrome [29] Multiple Sclerosis [30, 31] Concussion [32]
Measure addition or substitution	3	Include Timed Up and Go Test [31] Timed Up and Go Test instead of 10MWT [31, 33].
Construct	2	CPG assesses capacity but not performance [34]. Core Set provides an incomplete picture of community independence [35].
Setting	2	Limited utility in home health setting [36] Limited information about community independence [35].
Feasibility	2	Too many measures [37] 6MWT distance too long for MS [30]
Use of the core set	1	Need more guidance about how to combine two balance measures [38]
**Positive feedback**	14	
Core set	9	Core Set is an excellent assessment [39] Confirmed time points for administration [37, 39] Core Set supports high‐quality, value‐based care [37] Core Set is comprised of activities that relate to day‐to‐day activities [40] Core Set is useful for lower functioning individuals with concussion [32] Use of a self‐reported measure to assess balance confidence [41] Core Set is useful for data collection [42, 43]
Methodology	2	Used Core Set methodology for project [42] Core Set provides criteria for search and recommendations, graded evidence, suggested specific tools, recommended specific administration time points, and provides resources [18]
Specific measures	2	Use of the ABC [41] Use the FGA in addition to the BBS [27]
Resources	1	Resources provided to help end‐users with implementation [18]

Nine articles provided positive feedback about elements of the Core Set. The most common theme emphasised the value of the measures and recommendations provided in the Core Set for research and practice, describing it as a well‐designed assessment tool that offers clear points for administration, and indicating that it supports high‐quality and value‐based care, and captures functional activities [32, 37, 39, 40, 41, 42, 43]. Positive feedback also addressed the methodology, with authors acknowledging the Core Set's clear criteria for grading evidence, measure selection, timing of administration, and resources [18, 42]. Others emphasised the value of specific recommended measures [27, 41].

In addition to citation‐based dissemination indicators, online engagement metrics were collected. The Core Set had an Altmetric Attention Score of 60, placing it in the top 5% of all research outputs tracked by Altmetric [11]. When compared with research of a similar publication year, the CPG ranked #15,411 of 348,368 outputs, and it ranked #6 of 580 outputs published in the *Journal of Neurologic Physical Therapy* [11]. Among outputs of similar age within the same journal, it ranked #2 [11]. Altmetric has tracked ~29.3 million research outputs to date, and this CPG ranked #821,137 overall [11]. The CPG had 261 scholarly citations indexed across multiple databases (Scopus: 258; CrossRef: 163; PubMed Central: 113) [24]. It also received 2 citations in practice‐oriented CPG repositories and 1 policy citation [24]. In addition, 643 PlumX “captures” were recorded, indicating instances where users saved the document to a reference management or library system for later use. Of these captures, 604 were Mendeley readers, representing users who bookmarked the CPG in their Mendeley libraries and is viewed as an indicator of scholarly engagement [12]. The highest proportion of readers was identified within Nursing and Health Professions (55%), followed by Medicine and Dentistry (30%), Neuroscience (10%), and Sports and Rehabilitation (4%) [12]. Reader seniority data indicated representation at multiple levels of training and professional experience, including postgraduate trainees (69%), researchers (15%), professors (12%), and lecturers (5%) [12].

## 4 Discussion

This citation analysis provides a comprehensive overview of how the Core Set has been disseminated and used in literature since its publication. Between July 1, 2018, and July 31, 2024, 176 peer‐reviewed articles cited the Core Set, with most citations appearing in the introduction and methods sections, suggesting that it is being used both to frame research questions and to inform study design. Published analyses suggest that CPGs typically accrue modest citation rates. For example, Hong et al. found that radiology CPGs were cited 1–2 times in their first year, rising to 6–10 citations per year by years 3–5 [46]. Of the top 100 most‐cited neurorehabilitation papers published between 2005 and 2016, only two articles exceeded 1,000 total citations, while the majority achieved 200–800 citations over 10–15 years [47]. This range corresponds to approximately 15–40 citations per year, indicating that the Core Set's median of ~25 citations per year reflects a level of scholarly uptake that is comparable to that of highly influential research in neurorehabilitation. These findings suggest that the Core Set has been widely referenced and has contributed to scholarly discussion about measurement approaches in neurologic physical therapy, particularly in the United States but also internationally.

The reach and engagement indicators (accessed October 27, 2025) further demonstrate the broad visibility and scholarly attention associated with the Core Set within the rehabilitation and research communities. The Altmetric Attention Score of 60 places the Core Set in the top 5% of all research outputs tracked, reflecting substantial dissemination across digital platforms and professional communication channels [48, 49]. Additionally, more than 600 Mendeley readers and 643 PlumX captures suggest engagement among clinicians, educators, and researchers, as these metrics often reflect articles that are intentionally saved for future use [50, 51]. While these metrics indicate that the publication was widely accessed, shared, and saved for future reference, they do not indicate whether the document was read in full, considered useful, adopted, or implemented in practice. Future research should pair bibliometric and article‐level metrics with surveys, interviews, clinical audits, registry data, or electronic health record analyses to better evaluate awareness, perceived utility, adoption, and implementation of the Core Set in clinical practice. While 30% of article citations were coded to indicate that the Core Set influenced study design or selection of measures, very few explicitly described structured implementation in clinical practice. One explanation for this finding is a dissemination–implementation gap, in which evidence‐based CPGs are recognised within academic literature yet adopted more slowly in clinical practice [52]. Other work has shown that many CPGs are not designed while considering implementability, and they frequently omit features such as clear workflow integration guidance, user‐friendly formatting, and practical implementation supports [9, 52]. However, during the development of the Core Set, a dedicated Core Set Knowledge Translation Task Force worked in parallel to develop comprehensive implementation resources for the Core Set recommendations, including standardised administration protocols and clinician‐facing resources (e.g., standardisation procedures, patient report card, training videos), which have been accessed extensively online [23]. Evidence suggests that citation‐based metrics often undervalue clinical intervention work [53], thus the Core Set's influence may extend beyond what is captured in the literature. This suggests that implementation may be occurring more broadly than is reflected in peer‐reviewed publications, particularly given that many clinicians and health systems lack the time, training, or resources to design, evaluate, and publish implementation projects, even when this work is actively occurring [54]. Future impact evaluations should therefore extend beyond bibliometric data to include surveys, registry tracking, or collaborative implementation networks that can better capture the extent of Core Set adoption in practice.

The positive feedback from authors offers guidance for the Core Set revision by building on its valued features. Feedback highlighting the clarity of the literature search, grading criteria, and recommended administration procedures supports maintaining the same rigorous and transparent methodology for the revision. These features align with recommended implementability elements, including clarity, usability, and acceptability to clinicians and patients [55, 56, 57]. Authors also endorsed the overall structure and intent of the Core Set, indicating the value in having a defined group of outcome measures that could support research and clinical decision‐making. The recommended administration time points were viewed as practical and clinically meaningful, and the integration of self‐reported measures was appreciated for adding a patient‐centred dimension. Some authors also appreciated recommendations that span functional levels of balance, such as the inclusion of both the Berg Balance Scale to measure balance in sitting and standing and the Functional Gait Assessment to measure balance while walking. Research demonstrates that combining patient‐reported and observation‐based measures, as well as integrating multiple tools to assess a construct, provides a more comprehensive evaluation of function [58, 59]. Collectively, this feedback reinforces the importance of preserving the Core Set's structure, integration of performance and self‐report measures, and functional focus, which appear to be features that contribute to its perceived value and acceptance. Expanding on these components by strengthening the balance between patient‐reported and observation‐based measures and ensuring that included tools capture multiple aspects of function across levels of ability, could further enhance the Core Set's comprehensiveness and clinical relevance.

Constructive feedback in articles identified opportunities to strengthen applicability across functional levels and feasibility in clinical practice. Feedback on testing protocols suggests the need to evaluate the recommended standardisation procedures, such as walkway length, number of turns, and timing methods, so that clinicians and researchers can apply consistent protocols or justify deviations when necessary. This recommendation aligns with recent evidence demonstrating that variations in pathway length and turning frequency during the 6‐min Walk Test can significantly alter the distance walked [60, 61], underscoring the need to include standardisation procedures in the extraction protocol to fully evaluate them. Several authors noted that the Core Set primarily measures capacity (i.e., what individuals can do in a controlled setting), rather than performance, which reflects activity in their daily environments [62]. Distinguishing between capacity and performance is critical, as improvements observed in a clinic do not necessarily translate to functional gains in daily life [63]. Exploring a possible expansion of the Core Set to include measures that capture performance and community participation could strengthen its ecological validity and provide a more comprehensive assessment of recovery and function.

Finally, feedback related to feasibility and overlap of measures highlights the need to ensure that the Core Set remains practical for everyday clinical use. This aligns with research demonstrating that clinicians are more likely to use outcome measures when they are efficient to administer, easy to interpret, and compatible with clinical workflow [64, 65, 66]. To support this, the next revision should review and refine existing administration procedures and provide concise decision aids and workflow guidance, which are established strategies for improving the adoption of the Core Set in clinical settings [67]. Offering resources such as electronic documentation templates or clinical pathways may further enhance feasibility, acceptability, and appropriateness, which are key determinants of sustained use in practice [68]. Workflow fit, low burden, and clarity also facilitate sustainability of the implemented intervention [69, 70]. Collectively, this feedback suggests that the next revision should maintain the Core Set's methodological rigour while enhancing its usability and sustainability across diverse clinical settings.

Several limitations should be considered when interpreting these findings. First, this analysis was limited to peer‐reviewed, English‐language publications and intentionally excluded grey literature sources such as theses, institutional reports, and CPGs. As a result, the findings may not capture broader clinical, educational, institutional, quality improvement, or implementation‐related uses of the Core Set that were not reported in the peer‐reviewed literature. Second, while citation analysis provides an objective measure of scholarly influence, it does not necessarily reflect implementation in practice or education; citations may indicate awareness rather than direct use. Third, categorisation of citation context required interpretive judgement, which introduces potential for misclassification despite standardised coding and group review. Finally, publication and citation patterns evolve over time, so this 6‐year snapshot may not reflect ongoing trends in the Core Set's influence. Future studies could pair bibliometric analyses with registry data, electronic health record integration, or clinician and physical therapy consumer (i.e., patients, clients, caregivers) surveys to more comprehensively evaluate adoption and impact.

## 5 Conclusion

Overall, this citation analysis suggests that the Core Set has been widely cited across peer‐reviewed rehabilitation literature. The findings affirm support for the Core Set's structure, methodological rigour, and functional focus, while also highlighting opportunities for refinement. Existing literature highlights the value of maintaining standardised protocols, expanding applicability through measures that span levels of function, and reviewing and improving standardisation instructions and knowledge translation tools. These insights provide guidance for an evidence‐informed revision that balances scientific rigour with clinical practicality. Continued monitoring through both bibliometric and implementation‐focused evaluations will be essential to ensure that future iterations of the Core Set remain relevant, accessible, and effective in advancing standardised, evidence‐based neurologic rehabilitation.

## Author Contributions

**Jennifer L. Moore:** conceptualisation, investigation, funding acquisition, writing – original draft, writing – review and editing, visualisation, validation, methodology, software, formal analysis, project administration, resources, supervision, data curation. **Jane E. Sullivan:** conceptualisation, investigation, validation, methodology, data curation, supervision, formal analysis, software, funding acquisition, writing – original draft, writing – review and editing, visualisation, project administration, resources. **Marghuretta Bland:** conceptualisation, investigation, funding acquisition, writing – original draft, writing – review and editing, visualisation, validation, methodology, software, formal analysis, project administration, data curation, supervision, resources. **Wendy Romney:** conceptualisation, methodology, data curation, software, supervision, resources, project administration, formal analysis, validation, visualisation, writing – review and editing. **Elizabeth Anderl:** data curation, supervision, resources, project administration, formal analysis, software, methodology, validation, visualisation, writing – review and editing, writing – original draft, funding acquisition, investigation, conceptualisation. **Zeynep Gulen Erkoc:** software, investigation, validation. **Kirsten Potter:** data curation, supervision, resources, project administration, formal analysis, software, methodology, validation, visualisation, writing – review and editing, writing – original draft, funding acquisition, investigation, conceptualisation.

## Funding

The authors have nothing to report.

## Conflicts of Interest

Drs. Sullivan, Moore, and Potter were co‐authors of the 2018 CPG.

## Supporting information


Supporting File S1

Supporting File S2

Supporting File S3

## Data Availability

The data that support the findings of this study are available on request from the corresponding author. The data are not publicly available due to privacy or ethical restrictions.
