# Supplementary material for: Planning a Clinical Practice Guideline Revision for a Core Set of Outcome Measures in Adults Undergoing Neurologic Physical Therapy, Part 1: Assessing Dissemination and Scholarly Uptake
Source: Clin Public Health Guidel. 2026 Sep 6;3(4):e70086. doi: 10.1002/gin2.70086 (PMC13618606; doi:10.1002/gin2.70086)
Supplement: Supplementary file 1 [file GIN2-3-e70086-s001.docx]

Supplementary Table 2: Research Design of Study Citing the Core Set

| **Research Design** | **Citation Count**  **n=176** | **Percent** |
| --- | --- | --- |
| Psychometric study | 49 | 28% |
| Cohort study | 31 | 18% |
| Case study | 19 | 11% |
| Meta-analysis, Systematic Review | 16 | 9% |
| Opinion | 16 | 9% |
| Pilot/Feasibility | 12 | 7% |
| Clinical practice guidelines | 8 | 5% |
| Randomized Controlled Trial | 8 | 5% |
| Study protocol | 5 | 3% |
| Survey | 4 | 2% |
| Other* | 17 | 10% |

*Other (Count, Percent): Delphi Study (2, 1%), Mixed Methods (2, 1%), Qualitative (2, 1%), Citation Analysis (1, 1%), Cross-sectional Study (1, 1%)
