# Supplementary material for: Planning a Clinical Practice Guideline Revision for a Core Set of Outcome Measures in Adults Undergoing Neurologic Physical Therapy, Part 1: Assessing Dissemination and Scholarly Uptake
Source: Clin Public Health Guidel. 2026 Sep 6;3(4):e70086. doi: 10.1002/gin2.70086 (PMC13618606; doi:10.1002/gin2.70086)
Supplement: Supplementary file 2 [file GIN2-3-e70086-s002.docx]

Supplementary Table 1: Fields extracted for citation analysis.

**Fields**

Title

Authors

Institution of Corresponding Author

Location of Corresponding Author (country)

Publication Date

Geography: In what country was the study completed

Research Design

Areas of Practice

Diagnosis

Setting

Citation count

Sentences that include citation

Section of the paper where the CPG was cited

Which discipline used the measures, if applicable

Did the authors provide constructive feedback about the CPG? If yes, please describe

Did the authors provide positive feedback or praise the CPG? If yes, please describe
