# Supplementary material for: Planning a Clinical Practice Guideline Revision for a Core Set of Outcome Measures in Adults Undergoing Neurologic Physical Therapy, Part 1: Assessing Dissemination and Scholarly Uptake
Source: Clin Public Health Guidel. 2026 Sep 6;3(4):e70086. doi: 10.1002/gin2.70086 (PMC13618606; doi:10.1002/gin2.70086)
Supplement: Supplementary file 3 [file GIN2-3-e70086-s003.docx]

Supplementary Table 3: Location of all studies that cited Core Set and location of studies that cited Core Set related to citation context that was Directed, Integrated or Implemented in practice.

| **Country** | **Citation Count**  **179** |
| --- | --- |
| United States | 104 |
| Brazil | 7 |
| Japan | 6 |
| Italy | 5 |
| Norway | 5 |
| Australia | 4 |
| Canada | 4 |
| China | 4 |
| France | 4 |
| Germany | 3 |
| Iran | 3 |
| United Kingdom | 3 |
| India | 2 |
| Slovenia | 2 |
| Spain | 2 |
| Taiwan | 2 |
| Turkey | 2 |
| Other* | 17 |
| **Country for Citation Context Directed, Integrated or Implemented in Practice** | **Citation Count**  **57** |
| United States | 35 |
| Brazil | 6 |
| Germany | 3 |
| Italy | 2 |
| Japan | 2 |
| United Kingdom | 2 |
| Other† | 7 |

*Other (citation count of 1): Austria, Bahrain, Belgium, Denmark, Greece, Malaysia, Netherlands, Pakistan, Portugal, Saudia Arabia, Singapore, Sweden, Tunisia, and an International multi-site project.

†Other (citation count of 1): Austria, Canada, China, France, Spain, Sweden, Tunisia
